# Genetic variants associated with circulating MMP1 levels near matrix metalloproteinase genes on chromosome 11q21-22 in Taiwanese: interaction with obesity

**DOI:** 10.1186/1471-2350-14-30

**Published:** 2013-03-04

**Authors:** Hsuan-Li Huang, Semon Wu, Lung-An Hsu, Ming-Sheng Teng, Jeng-Feng Lin, Yu-Chen Sun, Yu-Lin Ko

**Affiliations:** 1 Department of Internal Medicine, Division of Cardiology, Buddhist Tzu Chi General Hospital, Taipei branch, 289 Jianguo Road, Xindian City, Taipei, 231, Taiwan; 2 Department of Life Science, Chinese Culture University, Taipei, Taiwan; 3 Department of Internal Medicine, The First Cardiovascular Division, Chang Gung Memorial Hospital, Taipei, Taiwan; 4 Chang Gung University College of Medicine, Taipei, Taiwan; 5 Department of Medical Research, Buddhist Tzu Chi General Hospital, Taipei branch, Taipei, Taiwan; 6 Department of Laboratory Medicine, Chang Gung Memorial Hospital, Taipei, Taiwan; 7 School of Medicine, Tzu Chi University, Hualien, Taiwan

**Keywords:** Matrix metalloproteinase 1, Genetic association study, Polymorphism, Haplotype, Interaction

## Abstract

**Background:**

MMP1 is implicated in the pathogenesis of atherothrombotic cardiovascular disease. We aimed to elucidate genetic determinants of inflammatory marker levels, including circulating MMP1, in Taiwanese, and their association with obesity.

**Methods:**

Five genetic polymorphisms around matrix metalloproteinase genes on chromosome 11q21-22 region were genotyped in 519 subjects.

**Results:**

After adjusting for clinical covariates, two polymorphisms were significantly associated with MMP1 levels, rs1799750 and rs495366, using an additive inheritance model (*P* = 1.5x10^-4^ and *P* = 2.57x10^-5^, respectively). Using dominant model, minor alleles of rs1799750 and rs495366 were associated with higher MMP1 levels (*P* = 1.3x10^-4^ and *P* = 1.95x10^-5^, respectively). In haplotype analysis, two haplotypes inferred from five SNPs (*A2GATA* and *A1GATG*) were associated with MMP1 levels (*P* = 5x10^-4^ and *P* = 8.47x10^-5^, respectively). Subgroup and interaction analysis revealed an association of rs1799750 and rs495366 with MMP1 levels only in non-obese subjects (*P* = 6.66x10^-6^ and *P* = 4.38x10^-5^, respectively, and interaction *P* = 0.008 for rs1799750). Haplotype interaction analysis also showed significant interaction for haplotype *A1GATG* (interaction *P* = 0.003).

**Conclusions:**

Genotypes/haplotypes around *MMP1* locus are associated with MMP1 levels in Taiwanese. Further, since genotypes/haplotypes near *MMP1* locus interact with obesity to set MMP1 levels, genetic determinants for MMP1 level may be different between obese and non-obese individuals.

## Background

Matrix metalloproteinases (MMPs) are a family of Zn^2+^-dependent endopeptidases capable of cleaving components of extracellular matrix, such as collagen, proteoglycans and elastin [1,2].

For cardiovascular diseases, MMPs have been identified in human atherosclerotic plaque shoulders and regions of foam cell accumulation and may contribute to plaque vulnerability [3,4]. Further, MMPs are suspected to play an important role in the pathogenesis of atherosclerosis, restenosis, and myocardial repair following myocardial infarction [3-5].

Matrix metalloproteinase 1 (MMP1), also called interstitial collagenase, is a proteolytic enzyme able to degrade types I, II, and III collagen, which are resistant to most proteinases [6,7]. Pathological studies have shown that MMP1 is expressed by macrophages in atherosclerotic plaques and MMP1 expression is significantly increased in vulnerable atherosclerotic plaques compared with stable plaques, particularly in shoulder regions and areas of foam cell accumulation in the plaques [8-11]. MMP1 is also implicated in the regulation of the platelet aggregation that follows plaque disruption which may result in acute coronary syndrome [12]. These findings suggested that MMP1 may play an important role in the pathogenesis of atherothrombotic cardiovascular disease.

Recent studies have shown an association of MMP1 levels with a wide variety of disorders, including polycystic kidney disease, rheumatoid arthritis, idiopathic pulmonary fibrosis, congestive heart failure, and acute myocardial infarction [13-17]. Promoter polymorphisms of the *MMP1* gene have also been associated with increased *MMP1* gene expression [18-20]. However, controversial results were noted between *MMP1* promoter polymorphisms, circulating MMP1 levels and the risk of cardiovascular disease [16,21-28]. Using a genome-wide association scan, Cheng et al. identified variants near *MMP* genes on chromosome 11q21-22 that were significantly associated with serum MMP1 levels [29].

We analyzed five closely linked SNPs located near *MMP* genes on chromosome 11q21-22 in an attempt to elucidate the genetic determinants of circulating MMP1 levels in Taiwanese and perform interaction analysis for obesity.

## Methods

### Subjects

A total of 519 Han Chinese subjects (264 men with a mean age of 43.9 ± 9.4 years and 255 women with a mean age of 45.8 ± 9.4 years) responded to a questionnaire on their medical history and lifestyle characteristics and were recruited during routine health examinations between Oct. 2003 and Sept. 2005 at the Chang Gung Memorial Hospital. The subjects underwent a physical examination that involved measurement of height, weight, waist and hip circumference, and blood pressure in the sitting position after 15 min of rest. Fasting blood samples were obtained from each subject. Exclusion criteria included a history of myocardial infarction, stroke or transient ischemic attack, cancer, and current renal or liver disease. The clinical characteristics and biometrics of the study population are summarized in Table 1.

**Table 1:** Baseline characteristics of study subjects

	**Total**	**Men**	**Women**	***P *value**
Number	519	264	255	
Age (years)	44.8 ± 9.4	43.9 ± 9.4	45.8 ± 9.4	0.023
Body mass index (kg/m^2^)	24.1 ± 3.4	24.8 ± 3.1	23.4 ± 3.6	<0.001
Current smokers (%)	19.8	35.2	3.9	<0.001
CRP (mg/L)	1.0 ± 1.4	1.1 ± 1.4	1.0 ± 1.3	0.071
Fibrinogen (mg/dL)	260.3 ± 66.6	257.7 ± 68.6	263.2 ± 64.6	0.348
sE-selectin (μg/L)	52.6 ± 25.7	59.8 ± 27.0	45.0 ± 22.0	<0.001
sP- selectin (ng/mL)	135.9 ± 114.1	149.2 ± 129.6	122.2 ± 94.0	0.004
SAA, (mg/L)	5.1 ± 10.1	5.4 ± 11.6	4.7 ± 8.4	0.485
sICAM1 (μg/L)	239.8 ± 113.2	243,8 ± 109.6	235.8 ± 116.8	0.281
sVCAM1 (μg/L)	488.0 ± 133.7	490.4 ± 154.2	485.4 ± 108.8	0.647
MMP1 (pg/mL)	486.9 ± 1213.4	355.1 ± 591.4	622.7 ± 1612.2	0.992
MMP2 (ng/mL)	126.4 ± 40.8	123.0 ± 41.9	129.9 ± 39.5	0.013
MMP9 (ng/mL)	142.1 ± 111.8	154.1 ± 112.8	129.7 ± 109.7	<0.001
MCP1 (pg/mL)	74.0 ± 61.0	80.9 ± 71.2	66.9 ± 47.4	0.006
sTNFRII (pg/mL)	3237.3 ± 922.3	3300.2 ± 966.8	3172.7 ± 871.3	0.116
IL6 (pg/mL)	4.0 ± 7.5	4.2 ± 8.8	3.8 ± 5.8	0.426

*P* value indicates male *vs.* female.

Obesity was defined as a body mass index (BMI) of 25 kg/m^2^, or more, according to the Asian criteria [30]. Current smokers were defined as those who smoked cigarettes regularly at the time of survey. All subjects provided written informed consent. The study was approved by the Ethics Committee of the Tzu-Chi Memorial Hospital.

### Genomic DNA extraction and genotyping

Genomic DNA was extracted, as previously reported [31,32]. Oligonucleotide primers were generated to amplify fragments of genomic DNA containing SNPs, as reported on the NCBI SNP database (http://www.ncbi.nlm.nih.gov/SNP). Five SNPs were analyzed due to previous reports showing an association with *MMP1* gene expression/level. Genotyping for SNPs rs11226373, rs1799750 and rs495366 were performed by polymerase chain reaction with restriction enzyme digestion. Genotyping for SNP rs514921 (C_632711_10) and rs1144393 (C_7492493_10) were performed using TaqMan SNP Genotyping Assays obtained from Applied Biosystem (ABI, Foster City, CA, USA). Genotyping data are shown in Additional file 1: Table S1.

### Laboratory examination and assays

Medical history and blood sampling were obtained, as previously reported [33]. Most markers, including serum C-reactive protein (CRP), serum amyloid A (SAA), soluble intercellular adhesive molecule (sICAM1), soluble vascular cell adhesive molecule (sVCAM1), soluble E-selectin (SELE), matrix metalloproteinase 2 (MMP2), and matrix metalloproteinase 9 (MMP9) were measured using a sandwich enzyme-linked immunosorbent assay (ELISA) developed in-house. All in-house kits showed good correlation when compared with commercially available ELISA kits [34-36]. Circulating plasma matrix metalloproteinase 1 (MMP1), Monocyte chemotactic protein 1 (MCP1), soluble P-selectin (SELP), soluble tumor necrosis factor receptor II (sTNFRII), and interleukin 6 (IL6) were measured using commercially available ELISA kits from R&D (Minneapolis, MN, USA). Mean intra-assay CVs from serum specimens were 7.1, 8.5, 2.1, 4.2, 4.1, 6.1 and 7.1%, and inter-assay CVs were 9.5, 8.1, 3.8, 6.8, 3.4, 8.8 and 9.1% for CRP, SAA, ICAM-1, sVCAM1, SELE, MMP2,and MMP9 levels, respectively. While the intra-assay CVs from plasma specimens were 3.8, 3.0, 2.2, 3.1 and 5.5%, and inter-assay CVs were 5.7, 8.8, 4.1, 4.5 and 8.2% for MCP-1, SELP, sTNFRII, IL6 and MMP1 levels, respectively.

### Statistical analysis

The chi-square test was used to test for comparing categorical variable of smoking. The clinical characteristics of the continuous variables were expressed as means ± SD and tested by two-sample *t*-test or ANOVA. A generalized linear model was used to analyze MMP1 level in relation to predictors of the investigated genotypes and confounders. CRP, SAA, IL6, ICAM1, VCAM1, SELE, SELP, MMP1, MMP2, MMP9, MCP1, and sTNFRII were logarithmically transformed prior to statistical analysis to adhere to a normality assumption. The Bonferroni method was used to correct for multiple comparisons where applicable. A value of *P* < 0.05, using two-sided tests, was considered statistically significant, whereas a value of corrected *P* < 0.01 for genotype and *P* < 0.005 for haplotype analysis, respectively, was considered significant after Bonferroni correction.

Interactions between each SNP, the level of MMP1, and obesity status were tested with two-way ANOVA. When interaction terms were significant, stratified analyses of the genetic variants of the genotypes (e.g., target genotypes affected by obesity) and MMP1 level were performed to further investigate interactive effects while controlling for other variables including age, gender, and smoking. Measures of haplotype frequencies were estimated using the expectation-maximization algorithm implemented in HelixTree Genetics Analysis software (Golden Helix, Bozeman, MT). In the haplotype analysis, Coefficients and *P* values were estimated based on haplotype trend regression analysis implemented in the HelixTree program. The selected haplotype was compared to all other unselected haplotypes. The analysis of deviation from Hardy-Weinberg equilibrium, estimation of linkage disequilibrium between polymorphisms, association of haplotypes with MMP1 level, and haplotype-obesity interaction were performed using the Golden Helix SVS Win32 7.3.1 software.

## Results

### Clinical and biochemical characteristics

A summary of demographic features, clinical profiles, and inflammatory biomarkers for the study participants (stratified by sex) is provided in Table 1. No significant deviation from the Hardy-Weinberg equilibrium was detected for the studied polymorphisms (*P* = 0.90, 0.76, 0.56, 1.00 and 0.95 for SNPs rs11226373, rs1799750, rs1144393, rs514921, and rs495366, respectively). Only SNPs rs1144393 and rs514921 were in strong pairwise linkage disequilibrium (Additional file 1: Figure S1).

### Associations of the MMP1 gene polymorphisms with circulation levels of MMP1

To determine whether the *MMP1* genotypes affected circulating inflammatory marker levels, 13 inflammatory markers were analyzed, including CRP, fibrinogen, SAA, IL6, sICAM1, sVCAM1, sP- selectin, sP- selectin, sTNFRII, MMP1, MMP2 MMP9, and MCP1. Our results showed that genetic variants in or around the *MMP1* gene were significantly associated with MMP1 levels in Taiwanese. After adjusting for clinical covariates, significant associations with MMP1 level were observed for two polymorphisms, rs1799750 and rs495366, using an additive inheritance model (*P* = 1.5x10^-4^ and *P* = 2.57x10^-5^, respectively). Furthermore, with dominant model, minor alleles of rs1799750 and rs495366 were found to be associated with a higher MMP1 level (*P* = 1.3x10^-4^ and *P* = 1.95x10^-5^, respectively). These associations remained statistically significant after multiple comparisons adjustment with Bonferroni method in additive and dominant model (Table 2). In contrast, there was no significant difference between the studied *MMP1* genotypes and levels of other inflammatory biomarkers (Additional file 1: Table S2).

**Table 2:** **Association between *MMP1 *genotype and MMP1 levels**

***MMP1 *genotype**	**MMP1 levels**	***P *value**	**Adjusted *P *value**
rs11226373 *AA*	536.6 ± 1333.4 (376)	0.111	0.555
*AG*	321.7 ± 632.8 (120)		
*GG*	838.0 ± 1898.8 (12)		
*AA*	536.6 ± 1333.4 (376)	0.036	0.180
*AG + GG*	368.6 ± 829.9 (132)		
rs1799750 *2G/2G*	323.2 ± 794.7 (206)	1.5x10^-4^	7.5 x10^-4^
*2G/1G*	543.9 ± 1294.1 (242)		
*1G/1G*	895.5 ± 1921.5 (58)		
*2G/2G*	323.2 ± 794.7 (206)	1.3x10^-4^	6.5 x10^-4^
*2G/1G + 1G/1G*	611.9 ± 1439.8 (300)		
rs1144393 *TT*	482.6 ± 1222.6 (428)	0.334	1
*TC*	552.0 ± 1238.8 (80)		
*CC*	380.8 (1)		
*TT*	482.6 ± 1222.6 (428)	0.360	1
*TC + CC*	549.9 ± 1231.1 (81)		
rs514921 *AA*	454.9 ± 1122.9 (405)	0.905	1
*AG*	667.9 ± 1601.6 (97)		
*GG*	336.4 ± 260.2 (6)		
*AA*	454.9 ± 1122.9 (405)	0.923	1
*AG + GG*	648.5 ± 1556.8 (103)		
rs495366 *AA*	299.7 ± 729.6 (174)	2.57x10^-5^	1.29 x10^-4^
*AG*	561.7 ± 1352.7 (242)		
*GG*	683.6 ± 1547.7 (91)		
*AA*	299.7 ± 729.6 (174)	1.95x10^-5^	9.75 x10^-5^
*AG + GG*	594.9 ± 1407.3 (333)		

*P* value, adjusted for age, sex, BMI, and smoking status.

Adjusted *P* values were computed by the Bonferroni method.

### MMP1 haplotypes and MMP1 level

Because single SNP regression demonstrated that multiple sites within the *MMP1* gene significantly affected MMP1 level, haplotypes were inferred to capture possible allelic associations. In the present investigation, 10 common haplotypes (≥ 1% frequency) were derived from five SNPs, accounting for 95.2% of all inferred haplotypes. In haplotype analysis, two haplotypes inferred from five SNPs (*A2GTAA* and *A1GTAG*) were found to be associated with MMP1 level (*P* = 5.02x10^-4^ and *P* = 8.47x10^-5^, respectively). The observed association between haplotypes (*A2GTAA* and *A1GTAG*) and MMP1 levels turn into borderline significant after multiple comparisons adjustment with Bonferroni method (Table 3).

**Table 3:** **Association of *MMP1 *locus haplotypes with MMP1 level**

	**SNP 1**	**SNP 2**	**SNP 3**	**SNP 4**	**SNP 5**	**Frequency**	**Coefficient**	**SE**	**t.stat**	***P *value**	**Adjusted *P *value**
H1	*A*	*2G*	*T*	*A*	*A*	38.56%	−0.2311	0.0660	−3.5029	5.02x10^-4^	0.005
H2	*A*	*1G*	*T*	*A*	*G*	17.47%	0.3304	0.0834	3.9635	8.47x10^-5^	8.47 x10^-4^
H3	*A*	*2G*	*T*	*A*	*G*	9.66%	0.1597	0.1103	1.4481	0.148	1
H4	*A*	*2G*	*T*	*G*	*A*	6.61%	−0.0533	0.1570	−0.3392	0.735	1
H5	*G*	*2G*	*T*	*A*	*A*	5.62%	−0.2479	0.1394	−1.7782	0.076	0.760
H6	*A*	*1G*	*C*	*A*	*G*	5.48%	0.1326	0.1440	0.9212	0.357	1
H7	*A*	*1G*	*T*	*A*	*A*	4.47%	0.1285	0.1697	0.7572	0.449	1
H8	*G*	*1G*	*T*	*A*	*G*	2.87%	−0.4000	0.2532	−1.5798	0.115	1
H9	*A*	*1G*	*T*	*G*	*G*	2.65%	0.2969	0.2400	1.2373	0.217	1
H10	*G*	*2G*	*T*	*A*	*G*	1.81%	−0.1032	0.2987	−0.3457	0.730	1

SNP1: rs11226373, SNP2: rs1799750, SNP3: rs1144393, SNP4: rs514921, SNP5: rs495366.

Coefficients and *P* values were estimated based on haplotype trend regression analysis implemented in the HelixTree program. The selected haplotype compared to all other unselected haplotypes; adjusted for age, sex, smoking, and BMI.

Adjusted *P* values were computed by the Bonferroni method.

### Interaction analysis

As presented in Table 4, after adjustment of clinical covariates, subgroup and interaction analysis revealed an association of rs1799750 and rs495366 with MMP1 levels only in non-obese subjects (*P* = 6.66x10^-6^ and *P* = 4.38x10^-5^, respectively, and interaction *P* = 0.008 for rs1799750). In Additional file 1: Table S3, a significant effect of obesity on the association between *MMP1* haplotypes and MMP1 level was also noted in haplotype interaction analysis after adjusting for age, gender, and smoking, with the association found predominantly in non-obese subjects (*P* = 2.29x10^-6^ for haplotype *A1GTAG* and *P* = 0.002 for haplotype *A2GTAA*, and interaction *P* = 0.003 for *A1GTAG*). After stringent Bonferroni correction for multiple interaction tests, borderline significance was also noted with H5 (*G2GTAA*) for non-obese subjects and H8 (*G1GTAG*) for obese subjects.

**Table 4:** **Interaction effect of obesity on the association between *MMP1 *genotypes and MMP1 levels**

	**Non-obese**					**Obese**					
**SNP**	**N**	**Coefficient**	**SE**	**t value**	***P *value**	**N**	**Coefficient**	**SE**	**t value**	***P *value**	**Interaction *P***
rs11226373	322	−0.1193	0.0543	−2.1944	0.029	186	0.0305	0.0696	0.4374	0.662	0.100
rs1799750	320	0.1844	0.0402	4.5816	6.66x10^-6^	186	0.0041	0.0539	0.0752	0.940	0.008
rs1144393	323	0.0324	0.0741	0.4368	0.663	186	0.1128	0.0937	1.2039	0.230	0.536
rs514921	323	0.0097	0.0600	0.1623	0.871	185	0.0147	0.0897	0.1642	0.870	0.983
rs495366	321	0.1630	0.0393	4.1443	4.38x10^-5^	186	0.0689	0.0472	1.4608	0.146	0.119

Additive genetic model; Coefficients and *P* values were performed using the linear regression model under the assumption of an additive effect of the minor allele; adjusted for age, sex, and smoking.

## Discussion

This investigation analyzed the association of genetic variants in or around the *MMP1* gene with inflammatory marker levels in Taiwanese. The genotypes/haplotypes of the SNPs studied were associated with MMP1 levels, but not with levels of 12 other inflammatory markers. Furthermore, we found that genotypes/haplotypes around the *MMP1* locus interacted with obesity to set the MMP1 level, with the association occurring predominantly in non-obese subjects. These results provided the first evidence that genetic determinants for MMP1 level may vary between obese vs. non-obese individuals.

The role of SNPs found in or around the *MMP1* gene in *MMP1* gene expression/MMP1 levels has recently been examined. Rutter et al. [18] first reported that a *1G/2G* polymorphism at -1607 bp (rs1799750) in the *MMP-1* promoter region with the *2G* allele creates an Ets binding site and augments transcription. Affara et al. [20] also revealed an increase in *MMP1* gene expression of the *2G* allele. Pearce et al. [19] reported two other promoter polymorphisms on the *MMP1* gene, the *-519A > G* (rs1144393) and *-340T > C* (rs514921) polymorphisms. *In vitro* assays showed that, compared with the *A*_*519*_*-T*_*−340*_ haplotype, the *A*_*−519*_*-C*_*−340*_ and *G*_*−519*_*-T*_*−340*_ haplotype had lower promoter activity, whereas the *G*_*−519*_*-C*_*−340*_ haplotype had greater promoter strength in driving gene expression in human macrophages. However, the association between *-519A > G* and *-340T > C* polymorphisms and MMP1 level has not been reported and the association between -1607 *1G/2G* polymorphism and MMP1 level has been controversial. Abd-Allah et al. [28] showed higher MMP1 levels with the *2G* allele of the *-1607 1G/2G* polymorphism in a diseased population; Chen et al. [17] revealed lower MMP1 level with *-1607 2G* allele in rheumatoid arthritic patients, while two other reports showed no evidence of the association [16,27] .

By using a genome-wide association scan, the strongest evidence of an association between *MMP1* SNPs and MMP1 level was provided by Cheng et al. [29]. In that study, variants near *MMP* genes on chromosome 11q21-22 were associated with serum MMP1 levels, with the peak association noted at rs495366 with lower MMP1 levels in subjects containing the minor *T* allele (equal to *A* allele in our genotypic data). We found results similar to those reported by Cheng et al. [29], i.e., we showed a significantly lower MMP1 level in the *A* allele of the rs495366. In addition, we also found incomplete linkage disequilibrium of the studied polymorphisms with the *A* allele of the rs495366 linked to *2G* allele of the -1607 *1G/2G* polymorphism, which was associated with higher *MMP1* gene expression in previous reports but lower MMP1 level in our study. These results suggested that the *-1607 1G/2G* polymorphism, although affecting *MMP1* gene expression*,* may not be the major determinant of MMP1 level in Taiwanese. Because the rs495366 polymorphism was located far away from the *MMP1* gene, it is likely that unidentified polymorphism(s) may be present between the studied SNPs that may affect the gene expression and result in different MMP1 levels.

### Interaction with obesity

The interplay between genetic and environmental factors is important in phenotype development of complex traits. We found an association of *MMP1* genotypes/haplotypes with MMP1 level predominantly in non-obese subjects. Our previous studies showed that the association between genetic variants and inflammatory marker levels occurred predominantly in non-obese subjects [37,38] or obese subjects [39,40]. It has been suggested that macrophage accumulation in adipose tissue has a central role in stimulating MMP1 and MMP3 production by preadipocytes, mediated by IL1β and TNFα, supporting the role of macrophages in stimulating tissue remodeling during adipose tissue expansion in obesity [41,42]. We found an association of *MMP1* genotypes/haplotypes with MMP1 level predominantly in non-obese subjects. It could be due to the small sample size in the obese group. Otherwise, the present results suggest the possibility that obese subjects who have high levels of many metabolic and inflammatory risk factors may mask the genetically associated changes.

### Association of MMP1 gene promoter SNPs and MMP1 levels

Several groups have shown an association between the *1G/2G* polymorphism in the *MMP1* gene and coronary artery disease [22,24], myocardial infarction [19,21], and peripheral artery disease [23]. In a meta-analysis of 39 studies with 42269 individuals, Li et al. [43] provide evidence that *MMP1 -1607* polymorphism has a mild to moderate effect on the incidence of coronary disease. In two meta-analyses with 12 and 33 studies, respectively, Liu et al. [44,45] also found an association between the *1G/2G* polymorphism at -1607 bp in the *MMP-1* promoter region and the risk of colorectal cancer and risk of metastasis in some cancers. Pearce et al. [19] reported two other promoter polymorphisms on the *MMP1* gene, the *-519A > G* and *-340T > C* polymorphisms, with haplotype effects on the risk of myocardial infarction. Han et al. [26] investigated the *-519A > G* and *–340T > C* promoter polymorphisms and found an association between the *MMP1* promoter genotypes/haplotypes and the risk of acute coronary syndrome in Chinese. However, Hlatky et al. [25] found no evidence of an association between circulating MMP1 levels and the risk of AMI. Thus, further studies may be necessary to elucidate the role of MMP1 SNPs/levels in the development of cardiovascular disease.

### Limitation of the study

The main limitation of our study was its modest sample size, which was not analyzed in any functional manner and showed only an arguable relationship with disease. However, significant differences were noted even when Bonferroni correction was stringently applied for multiple tests. The replication of our results using a second cohort, especially with a larger sample size and a different ethnic population, would strengthen our analysis. In addition, the cross-sectional nature of the present study limits our ability to infer a causal relation between MMP1 variants, MMP1 level, and various inflammation-related disorders.

## Conclusions

In conclusion, we found that the association between *MMP1* gene polymorphisms and MMP1 level was dependent on obesity status. The polymorphism(s) that affected both *MMP1* gene expression and MMP1 level require further study. Because our evidence suggested an association between *MMP1* gene polymorphisms and MMP1 levels, the discovery of causative unidentified polymorphisms will be important in elucidating the mechanism of MMP1 on cancer and atherosclerotic cardiovascular disease.

## Abbreviations

SNPs: Single nucleotide polymorphisms; BMI: Body mass index; CRP: C-reactive protein; SAA: Serum amyloid A; IL6: Interleukin 6; sICAM1: Soluble intercellular adhesive molecule 1; sVCAM1: Soluble vascular cell adhesive molecule 1; sE-selectin: Soluble E-selectin; sP- selectin: Soluble P-selectin; MMP1: Matrix metalloproteinase 1; MMP2: Matrix metalloproteinase 2; MMP9: Matrix metalloproteinase 9; MCP1: Monocyte chemotactic protein 1; sTNFRII: Soluble tumor necrosis factor receptor II.

## Competing interests

The authors declare that they have no competing interests.

## Authors’ contributions

H-L H participated in genotyping, performed statistical analysis and drafted the manuscript. S W prepared the DNA samples, participated in genotyping, statistical analysis and drafted the manuscript. L-A H participated in sample collection and statistical analysis. M-S T prepared the DNA samples and participated in genotyping. J-F L prepared the DNA samples. Y-C S participated in ELISA assay. Y-L K supervised the study and revised the manuscript. All authors read and approved the final manuscript.

## Pre-publication history

The pre-publication history for this paper can be accessed here:

http://www.biomedcentral.com/1471-2350/14/30/prepub

## Supplementary Material

Additional file 1 The association between MMP1 haplotypes and MMP1 levels. Click here for file
