# Supplementary material for: Genetic variants associated with circulating MMP1 levels near matrix metalloproteinase genes on chromosome 11q21-22 in Taiwanese: interaction with obesity
Source: BMC Med Genet. 2013 Mar 4;14:30. doi: 10.1186/1471-2350-14-30 (PMC3599409; doi:10.1186/1471-2350-14-30)

Supplement Table 1. Primer sequences and restriction enzyme (RE) used for *MMP-1* gene polymorphisms

| SNP number | Primer sequence | PCR size and RE | Allele | Minor allele  (frequency) | Position |
| --- | --- | --- | --- | --- | --- |
| rs11226373 | (F)5’-TAGGTTCTCTAGGACAGCAGAA-3’  (R)5’-ATGACAGCCATCATACTAGTGG-3’ | 219, HpyCH4IV | *A/G* | *G* (0.14) | 103839449 |
| rs1799750 | (F)5’-AGTATATCTGCCACTCCTTGACTTTTAAAA-3’  (R)5-ATCTTGGATTGATTTGAGATAAGTCAGATC-3’ | 289, BamHI | *1G/2G* | *G* (0.35) | 102670496 |
| rs1144393 | TaqMan SNP Genotyping Assays(C_7492493_10) |  | *C/T* | *C* (0.08) | 102669409 |
| rs514921 | TaqMan SNP Genotyping Assays(C_632711_10) |  | *A/G* | *G* (0.11) | 102669230 |
| rs495366 | (F)5’-GCTTGGCATATAATGAGTGCTT-3’  (R)5’-TCAATGTTCTGAGACCACATCA-3’ | 303, SfcI | *A/G* | *G* (0.42) | 102200318 |

Supplement Table 2. Serum levels of inflammatory markers stratified by *MMP1* genotype

| rs Number | Biomarker | Genotypes |  |  | *P** |
| --- | --- | --- | --- | --- | --- |
| rs11226373 |  | AA | AG | GG |  |
|  | SAA (mg/L) | 5.19±11.44(369) | 4.74±5.04(119) | 3.07±3.14(11) | 0.748 |
|  | Fibrinogen (mg/dL) | 261.83±66.77(377) | 258.83±65.72(120) | 238.46±65.95(12) | 0.250 |
|  | IL6 (pg/mL) | 4.28±8.58(359) | 3.18±2.88(115) | 3.97±5.95(12) | 0.860 |
|  | sVCAM1 (μg/L) | 485.71±138.11(372) | 497.83±122.55(120) | 482.83±141.11(12) | 0.487 |
|  | sICAM1 (μg/L) | 242.66±118.23(372) | 230.03±102.67(120) | 276.11±70.05(12) | 0.788 |
|  | sE-selectin (μg/L) | 53.45±26.40(370) | 50.19±23.59(120) | 55.91±28.30(12) | 0.216 |
|  | sP- selectin (ng/mL) | 139.01±120.20(376) | 131.36±97.64(120) | 130.71±110.39(12) | 0.639 |
|  | MMP2 (ng/mL) | 124.98±37.73(372) | 131.14±50.72(120) | 120.68±28.66(12) | 0.526 |
|  | MMP9 (ng/mL) | 140.96±103.75(370) | 146.87±136.44(116) | 148.23±126.28(12) | 0.866 |
|  | MCP1(pg/mL) | 76.67±64.20(376) | 67.61±53.75(120) | 73.97±37.59(12) | 0.318 |
|  | sTNFRII (pg/mL) | 3240.41±911.45(376) | 3263.51±999.30(120) | 3296.86±558.90(12) | 0.639 |
|  | CRP (mg/L) | 1.07±1.46(377) | 0.98±0.95(120) | 0.89±1.13(12) | 0.790 |
| rs1799750 |  | GG | G- | -- |  |
|  | SAA (mg/L) | 4.62±7.22(203) | 5.13±10.41(238) | 6.27±16.48(56) | 0.779 |
|  | Fibrinogen (mg/dL) | 264.53±66.14(206) | 255.09±67.96(243) | 271.66±59.49(58) | 0.774 |
|  | IL6 (pg/mL) | 4.51±9.35(196) | 3.57±5.79(231) | 4.15±7.23(57) | 0.864 |
|  | sVCAM1 (μg/L) | 484.82±107.59(204) | 480.75±111.77(240) | 535.03±251.61(58) | 0.427 |
|  | sICAM1 (μg/L) | 244.96±109.28(204) | 234.03±98.33(240) | 255.33±174.30(58) | 0.394 |
|  | sE-selectin (μg/L) | 51.20±23.84(203) | 53.02±23.68(239) | 57.21±38.19(58) | 0.230 |
|  | sP- selectin (ng/mL) | 122.21±95.70(206) | 149.44±129.21(242) | 140.84±110.12(58) | 0.106 |
|  | MMP2 (ng/mL) | 126.71±41.62(204) | 123.97±40.06(240) | 135.46±42.78(58) | 0.391 |
|  | MMP9 (ng/mL) | 142.49±112.88(202) | 136.64±107.77(237) | 170.30±128.14(57) | 0.423 |
|  | MCP1(pg/mL) | 82.40±75.12(206) | 68.97±51.63(242) | 69.51±39.97(58) | 0.124 |
|  | sTNFRII (pg/mL) | 3322.20±986.91(206) | 3205.11±890.68(242) | 3157.74±837.16(58) | 0.155 |
|  | CRP (mg/L) | 1.09±1.34(206) | 1.05±1.32(243) | 0.87±1.53(58) | 0.046 |
| rs514921 |  | AA | AG | GG |  |
|  | SAA (mg/L) | 4.97±9.99(398) | 5.40±11.11(95) | 3.94±2.16(6) | 0.523 |
|  | Fibrinogen (mg/dL) | 259.14±64.38(405) | 266.93±75.19(98) | 259.15±50.88(6) | 0.142 |
|  | IL6 (pg/mL) | 3.90±7.47(390) | 4.50±8.17(90) | 2.06±1.25(6) | 0.659 |
|  | sVCAM1 (μg/L) | 488.56±138.18(401) | 489.31±120.68(97) | 463.17±82.05(6) | 0.957 |
|  | sICAM1 (μg/L) | 242.98±113.78(401) | 234.37±115.54(97) | 176.78±75.26(6) | 0.186 |
|  | sE-selectin (μg/L) | 53.12±26.45(399) | 51.65±23.41(97) | 46.18±17.09(6) | 0.640 |
|  | sP- selectin (ng/mL) | 135.67±110.44(405) | 145.88±133.98(97) | 99.95±58.71(6) | 0.817 |
|  | MMP2 (ng/mL) | 126.18±41.64(401) | 123.71±33.37(97) | 173.67±79.44(6) | 0.286 |
|  | MMP9 (ng/mL) | 141.84±114.66(397) | 146.40±106.70(95) | 124.61±54.58(6) | 0.778 |
|  | MCP1(pg/mL) | 73.43±59.24(405) | 79.76±71.13(97) | 49.57±33.48(6) | 0.935 |
|  | sTNFRII (pg/mL) | 3247.49±941.39(405) | 3205.96±828.57(97) | 3793.52±1305.23(6) | 0.458 |
|  | CRP (mg/L) | 1.03±1.34(405) | 1.11±1.43(98) | 0.45±0.40(6) | 0.578 |
| rs1144393 |  | CC | CT | TT |  |
|  | SAA (mg/L) | 2.90(1) | 6.83±17.44(79) | 4.70±8.07(420) | 0.813 |
|  | Fibrinogen (mg/dL) | 230.70(1) | 259.76±71.03(77) | 260.74±65.64(429) | 0.751 |
|  | IL6 (pg/mL) | 9.50(1) | 3.64±6.09(77) | 4.03±7.80(409) | 0.290 |
|  | sVCAM1 (μg/L) | 460.00(1) | 487.85±112.98(80) | 489.05±138.41(424) | 0.628 |
|  | sICAM1 (μg/L) | 283.20(1) | 229.93±95.42(80) | 242.22±117.05(424) | 0.298 |
|  | sE-selectin (μg/L) | 67.77(1) | 53.65±29.05(80) | 52.52±25.14(428) | 0.969 |
|  | sP- selectin (ng/mL) | 168.90(1) | 140.47±96.35(80) | 136.39±118.07(428) | 0.328 |
|  | MMP2 (ng/mL) | 106.80(1) | 124.33±49.24(80) | 126.80±39.39(424) | 0.365 |
|  | MMP9 (ng/mL) | 241.38(1) | 147.56±113.01(79) | 141.14±112.51(419) | 0.687 |
|  | MCP1(pg/mL) | 46.20(1) | 69.90±40.59(80) | 75.24±64.62(428) | 0.882 |
|  | sTNFRII (pg/mL) | 2947.50(1) | 3244.44±975.67(80) | 3246.63±917.11(428) | 0.737 |
|  | CRP (mg/L) | 0.19(1) | 1.11±1.63(80) | 1.03±1.29(429) | 0.474 |
| rs495366 |  | AA | AG | GG |  |
|  | SAA (mg/L) | 4.40±6.80(171) | 5.24±10.89(237) | 5.69±13.12(90) | 0.559 |
|  | Fibrinogen (mg/dL) | 263.22±68.39(174) | 255.14±66.06(242) | 270.60±63.03(92) | 0.938 |
|  | IL6 (pg/mL) | 4.99±9.03(164) | 3.34±6.86(232) | 3.97±6.19(89) | 0.162 |
|  | sVCAM1 (μg/L) | 492.25±113.14(172) | 477.06±111.98(239) | 511.67±205.40(92) | 0.682 |
|  | sICAM1 (μg/L) | 241.27±121.39(172) | 235.45±94.35(239) | 251.03±143.10(92) | 0.625 |
|  | sE-selectin (μg/L) | 51.74±25.45(171) | 50.38±21.70(238) | 60.85±33.75(92) | 0.092 |
|  | sP- selectin (ng/mL) | 137.66±122.04(174) | 137.83±115.10(242) | 134.53±100.82(91) | 0.946 |
|  | MMP2 (ng/mL) | 128.00±40.35(172) | 121.86±35.02(239) | 134.89±53.94(92) | 0.551 |
|  | MMP9 (ng/mL) | 139.35±96.65(172) | 136.36±114.58(234) | 164.98±132.28(91) | 0.439 |
|  | MCP1(pg/mL) | 79.16±71.05(174) | 71.78±57.82(242) | 72.99±50.18(91) | 0.411 |
|  | sTNFRII (pg/mL) | 3310.94±910.24(174) | 3173.15±876.67(242) | 3316.05±1067.36(91) | 0.419 |
|  | CRP (mg/L) | 1.04±1.32(174) | 1.01±1.23(242) | 1.14±1.69(92) | 0.450 |

CRP, C-reactive protein; HDL, high-density lipoprotein; LDL, low-density lipoprotein; SAA, serum amyloid A; IL6, interlukine 6; sICAM1, soluble intercellular adhesive molecule 1; sVCAM1, soluble vascular cell adhesive molecule 1; sE-selectin, soluble E-selectin ; sP- selectin, soluble P-selectin; MMP1, matrix metalloproteinase 1; MMP2, matrix metalloproteinase 2; MMP9, matrix metalloproteinase 9; MCP1, ; sTNFRII, soluble tumor necrosis factor receptor II.

Continuous variables are presented as mean ± SD. SAA, CRP, sICAM1, sVCAM1, sP-selectin, MMP1, MCP1 and IL6 values were logarithmically transformed before statistical testing to meet the assumption of normal distributions; however, the untransformed data are shown.

*P**, adjusted for age, sex, BMI and smoking

Supplement Table 3. Interacted effect of obesity on the association between *MMP1* haplotypes and MMP1 levels

|  | Non obese |  |  |  | Obese |  |  |  |  |
| --- | --- | --- | --- | --- | --- | --- | --- | --- | --- |
|  | Coefficient | SE | t value | P value | Coefficient | SE | t value | P value | Interaction *P* |
| H1 | -0.2663 | 0.0861 | -3.0926 | 0.002 | -0.1692 | 0.1044 | -1.6218 | 0.107 | 0.463 |
| H2 | 0.5293 | 0.1099 | 4.8156 | 2.29x10^-6^ | 0.0350 | 0.1273 | 0.2750 | 0.784 | 0.003 |
| H3 | 0.1015 | 0.1476 | 0.6875 | 0.492 | 0.2137 | 0.1675 | 1.2758 | 0.204 | 0.600 |
| H4 | -0.0799 | 0.1906 | -0.4191 | 0.675 | 0.0606 | 0.2866 | 0.2115 | 0.833 | 0.751 |
| H5 | -0.5020 | 0.1791 | -2.8037 | 0.005 | 0.1808 | 0.2196 | 0.8233 | 0.411 | 0.019 |
| H6 | 0.0893 | 0.1852 | 0.4821 | 0.630 | 0.2414 | 0.2323 | 1.0395 | 0.300 | 0.680 |
| H7 | 0.2733 | 0.2159 | 1.2657 | 0.207 | -0.1899 | 0.2812 | -0.6751 | 0.500 | 0.227 |
| H8 | -0.0373 | 0.3178 | -0.1173 | 0.907 | -1.1912 | 0.4208 | -2.8309 | 0.005 | 0.036 |
| H9 | 0.4057 | 0.2910 | 1.3941 | 0.164 | 0.0482 | 0.4369 | 0.1103 | 0.912 | 0.547 |
| H10 | -0.8460 | 0.4345 | -1.9471 | 0.052 | 0.6631 | 0.4001 | 1.6575 | 0.099 | 0.016 |

*P*, adjusted for age, sex, and smoking

Supplement Figure 1. Linkage disequilibrium between *MMP1* genetic polymorphisms


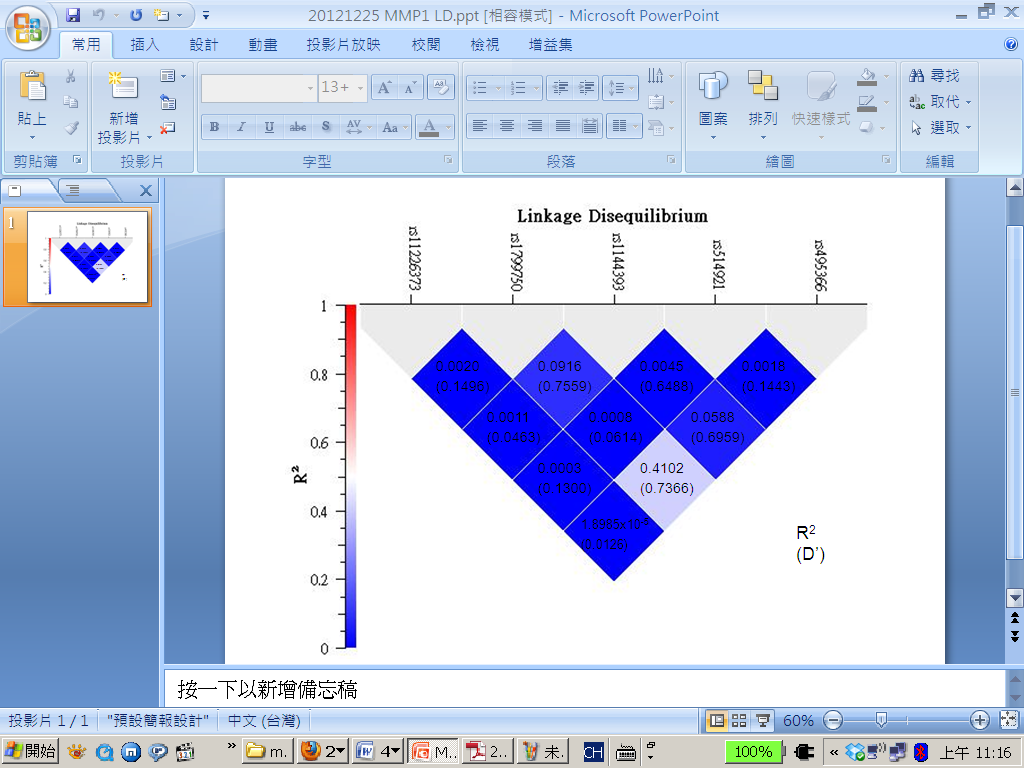

Supplement: Additional file 1 — The association between MMP1 haplotypes and MMP1 levels. [file 1471-2350-14-30-S1.doc]
